# Supplementary material for: Computer-Based Simulators in Pharmacy Practice Education: A Systematic Narrative Review
Source: Pharmacy (Basel). 2023 Jan 2;11(1):8. doi: 10.3390/pharmacy11010008 (PMC9844304; doi:10.3390/pharmacy11010008)
Supplement: Supplementary file 1 [file pharmacy-11-00008-s001.zip › pharmacy-2063546-supplementary.pdf]

pharmacy MDPI

Supplementary material

Table S1. *Characteristics of the included studies*

Study (Authors and year)	Country	Study Focus	Data collection methods/ Evaluation methods	Participants	Computer-based simulation platform	Features
Al-Dahir, Bryant, Kennedy, & Robinson, 2014 [51]	USA	To evaluate computer simulator as a learning method	Knowledge-based pre-post- Multiple-choice questions (MCQs)<br>Evaluative survey - Likert scale	Pharmacy undergraduates at one university.	DecisionSim (Formerly vpSim) https://www.kynectiv.com/platform (Active)	Automated marking/grading<br>Immediate detailed feedback<br>Single user<br>Different setting environment (Pharmacy + Interdisciplinary)<br>Support sharing scenarios<br>Video, Audio, and Imagery (Web-based)
Neal Benedict, 2010 [36]	USA	Incorporating effective and active-learning strategies	Knowledge based questions.<br>SOAP (Subjective-objective assessment-plan).<br>Post course evaluative survey	Pharmacy undergraduates at one university.		
Benedict, Schonder, & McGee, 2013 [28]	USA	To assess the effectiveness of computer simulation and promote self-directed learning	Pre and post knowledge quiz<br>Final exam scores<br>Post-course evaluative survey (Likert scale)	Pharmacy undergraduates at one university		
Lichvar et al., 2016 [66]	USA	To design and evaluate the integration of a virtual patient activity And study an effective active-learning strategy.	Pre and post knowledge quiz<br>End-of-year exam (To assess knowledge retention)<br>evaluative survey (Likert scale and open-ended questions)	Pharmacy undergraduates at one university.		
Olin & Cole, 2015 [55]	USA	To describe computer-based clinical	Evaluative survey (Likert scale)	Pharmacy undergraduate		

		simulation cases (CBCSCs) and review student perceptions of this learning strategy.		s at two campuses		
Michael A. Smith, Mohammad, & Benedict, 2014 [4]	USA	To assess student satisfaction and learning of course objectives	The post-course survey, and a pre-simulation and post-simulation tests were used to assess student learning	Pharmacy undergraduates at one university		
Michael A Smith, Siemianowski, & Benedict, 2016 [29]	USA	To expand the use of virtual patients by sharing case scenarios across two schools	Pre and post-tests assessed student learning, data collected regarding the development and sharing of cases and the survey was conducted to measure faculty perception and student satisfaction	Pharmacy undergraduates and 3 educators at two schools.		
Ambroziak, Ibrahim, Marshall, & Kelling, 2018 [12]	USA	To evaluate a computer simulator and assess the dispensing knowledge	Post-course survey (with open and closed-ended questions)	Pharmacy undergraduates at one university.	MyDispense<br>https://info.mydispense.monash.edu/<br>(Active)	Automated marking/grading<br>Immediate detailed feedback<br>Single user<br>One pharmacy setting<br>Support sharing scenarios<br>Static images, Text, and Patient notes (Web-based)
Ferrone, Kebodeaux, Fitzgerald, & Holle, 2017 [41].	USA	To evaluate computer simulator and assess the dispensing knowledge and student perception	post-simulation survey, (Likert scale and open-ended questions)	Pharmacy undergraduates at three Universities		
Mak, Fitzgerald, Holle, Vordenberg, & Kebodeaux, 2021 [11].	Australia	Improve student learning	A commentary from authors and developers of the tool to share suggestions for using "MyDispense" in the didactic and experiential settings to meet	Pharmacy undergraduates		

			educational outcomes and the challenges of virtual simulation.			
McDowell, Jenny Styles, Kim Sewell, Keith Trinder, Peta Marriott, Jennifer Maher, Sheryl Naidu, Som, 2016 [54].	Australia	To develop and evaluate a computer simulator and assess student knowledge.	Analysis of student examination results and post-course survey.	Pharmacy undergraduates at one university.		
Shin, Tabatabai, Boscardin, Ferrone, & Brock, 2018 [43]	USA	To demonstrate the feasibility of integrating computer simulation and assess student perceptions	pre- and post-tests in class and three surveys (for each phase and overall experience)	Pharmacy undergraduates at one university		
Tai, Ming-Hei Rida, Nada Klein, Kristin C Diez, Heidi Wells, Trisha Kippes, Kellie Walker, Paul C Vordenberg, Sarah E, 2020 [21].	USA	Assess student perception and knowledge in introductory pharmacy practice experiences (IPPEs)	Post-course surveys were conducted by students. Also, preceptors were asked to complete a survey.	Pharmacy undergraduates and preceptors at one university		
Richardson, Chapman, & White, 2019 [59].	different European countries	To develop and show proof of concept of the computer-based simulation.	a blend of formal meetings, distance evaluation (Using formal forms and email feedback).<br><br>Evaluation forms were a mixture of quantitative and qualitative questions.	A steering group of European pharmacists provided data for computer simulation development	Keele virtual patient (Keele University) https://www.keelevp.com/<br><br>(Active)	Grading was not offered (but suggested by users)<br><br>Immediate detailed feedback<br><br>Single user<br><br>Different setting environment (Pharmacy + Interdisciplinary)

Thompson, Jessica White, Simon Chapman, Stephen, 2020 [13].	UK	To evaluate perceptions on the integration, of computer simulator.	Telephone interviews (Semi-structured) to discuss the students' perspectives on the use of the virtual patient or non-interactive case studies	Pharmacy undergraduate (pre-registration) at one university		Support sharing scenarios<br><br>Interactive animated avatar, Embedded voice and Dynamic emotions, Patient notes and Dialogue choices options
Thompson, Jessica White, S. Chapman, S, 2020 [44].	UK	To evaluate virtual patient (VP) and non-interactive (NI) case studies, concerning knowledge, skill, and confidence development.	Pre- and post-knowledge quiz.<br><br>questionnaire (Likert scale and open-ended questions).	Pharmacy students (preregistered) at one university		
Bracegirdle & Chapman, 2010 [58].	UK	To demonstrate the computer simulator design and report the integration experience	collective feedback	Pharmacy undergraduates at one university		
S. B. Duffull & Peterson, 2020 [57].	New Zealand	to evaluate students' perceptions using the computer simulation	Post-course evaluative survey. (Likert scale)	Pharmacy undergraduates at one university		Automated grading<br><br>Immediate feedback<br><br>Single user<br><br>Different setting environment (Pharmacy + Interdisciplinary)<br><br>Support sharing scenarios<br><br>Static images, Audio, Text, and Patient notes (Web-based)
Duffull, Stephen. Peterson, A. K. Chai, Bill Cho, Frasier Opoku, Justice Sissing, Tasia Smith, Daniel Tongskul, Tran Wilby, K., 2020 [56].	New Zealand	To evaluate the use of the computer simulation in interprofessional education (IPE)	A post-course questionnaire, an interview, and a review of students' SimPHARM log files.	-Medical and pharmacy undergraduates at one university.	SimPharm<br><br>https://www.simpharm.com/<br><br>(Active)	
Loke, Swee-Kin	New Zealand	To evaluate the learning method	Audio recordings and	Pharmacy undergraduate		

Tordoff, June Winikoff, Michael McDonald, Jenny Vlugter, Peter Duffull, Stephen, 2010 [32]		using a computer simulator and students' perception	observations in class<br>Focus groups	s at one university.		
Wright, Daniel F. B. Duffull, Stephen B. Wilby, Kyle J. Peterson, Aynsley K. Anakin, Megan G.., 2020 [45].	New Zealand	To evaluate knowledge and skills delivered by the computer simulator	A pre-and post-intervention crossover study design	Practising pharmacists.		
Bravo, Marie Jam<br>So, Miranda Natsheh, Cindy<br>Tait, Gordon Austin, Zubin Cameron, Karen., 2019 [60].	Canada	To assess students' perceptions using a computer simulation	a semi-structured interview conducted by VIC's project team members.<br><br>The interviews were audio-recorded, transcribed, and coded for extracting themes.	Pharmacy undergraduates from one university	Virtual Interactive Case System<br><br>http://pie.med.utoronto.ca/VIC/VIC_content/VIC_pharm.html<br><br>(Active)	Automated marking/grading<br><br>Immediate detailed feedback<br><br>Single user<br><br>Different setting environment (Pharmacy + Interdisciplinary)<br><br>Support sharing scenarios<br><br>Static image and Text With limited choices option (Web-based form)
Dahri, Karen MacNeil, Kimberley Chan, Fong Lamoureux, Emilie Bakker, Mattie Seto, Katherine Yeung, Janice., 2019 [35].	Canada	To investigate students' perceptions towards computer simulation	Post-course survey and focus group participation	Pharmacy undergraduates at one university		
Bindoff, Ivan Ling, Tristan	Australia	To present and evaluate the computer	Pre and post knowledge quiz	Pharmacy undergraduate	Pharmacy Simulator	Automated marking/grading

Bereznicki, Luke Westbury, Juanita Chalmers, Leanne Peterson, Gregory Ollington, Robert., 2014 [16].		simulator's effectiveness and actively engaging learning experience	Evaluative survey (Likert scale and open-ended questions)	s from one university	https://www.pharmacysim.com/ <br>(Active)	Immediate detailed feedback <br>Single user <br>In more than one pharmacy setting <br>Support sharing scenarios <br>Interactive animated avatars, Embedded voice and Dynamic emotions, Patient notes and Dialogue choices options
Tait, Lauren Lee, Kenneth Rasiah, Rohan Cooper, Joyce M. Ling, Tristan Geelan, Benjamin Bindoff, Ivan., 2018 [67].	Australia	To investigate perception and experience using computer simulation	A post-simulation questionnaire.	Master of Pharmacy students at one university		
Zary, Johnson, Boberg, & Fors, 2006 [38].	Sweden	To evaluate a computer simulator	Post-course surveys and on-site observations were performed when possible. <br>(Likert scale and free text Comments)	Medicine, dental and pharmacy undergraduates	Web-based Simulation of Patients (Web-SP).	Automated marking/grading <br>Immediate detailed feedback <br>Single user <br>Different setting environment (Pharmacy + Interdisciplinary) <br>Support sharing scenarios <br>Video, Audio, and Imagery and/or free text input.
J. L. Marriott, 2007 [52].	Australia	To assess and evaluate a computer simulator's use as a learning tool	Post-assessment survey) to determine the	Pharmacy undergraduates at one School/University	A purpose-designed computer program	No marking/grading <br>Delayed detailed feedback

			program's ease of use and its perceived value.			Single user<br><br>Different setting environment (Pharmacy + Interdisciplinary)<br><br>Shareable scenarios: no information<br><br>Preselected options and/or text.
J. L. Marriott, 2007 [53].	Australia	To assess and evaluate a computer simulator's use as a learning tool	Review of the software design, features, and functions	Pharmacy undergraduates at one School/University		
Caylor, Aebersold, Lapham, & Carlson, 2015 [40].	USA	to examine the use and effectiveness of the computer simulation in multi-professional learning	Pre and post IPE Perception Scale (Likert scale)<br><br>Pre and post teamwork Questionnaire (Likert scale)<br><br>Team Performance Observation Tool – Faculty members<br><br>Post-course evaluative survey. (Likert scale and open-ended Q's)	Undergraduates from nursing, medicine, and pharmacy at same University	Second Life	Need faculty marking/grading<br><br>Immediate detailed feedback<br><br>Multiple users<br><br>Different setting environment (Pharmacy + Interdisciplinary)<br><br>Shareable scenarios – not supported<br><br>Animation based and voice discussion through avatars
Benedict & Schonder, 2011 [15].	USA	To implement and assess the effectiveness of a computer simulation in teaching	Pre and post knowledge quiz<br><br>Final exam scores<br><br>Post-course evaluative survey (Likert scale)	Pharmacy undergraduates from one university	PharmaCAL	Automated marking/grading<br><br>Immediate detailed feedback<br><br>Single user<br><br>In one pharmacy setting<br><br>Sharing scenarios – not supported<br><br>- Development: University developed<br><br>- Output: No info<br>- Input: Preselected options.

Chaikoolvatana & Goodyer, 2003 [61].	UK	To validate a computer simulator design and its use for learning	Care plan developed by students.<br><br>Face-to-face consultation<br><br>Post-course evaluative survey (Likert scale)	-Pharmacy undergraduates at one university	Multimedia case history program (MCHP)	Need faculty marking/grading<br><br>Immediate detailed feedback<br><br>Multiple users<br><br>In more than one pharmacy setting<br><br>Sharing scenarios – not supported<br><br>Video, Audio, and Imagery and text-based.
Cláudio, Carmo, Pinto, Cavaco, & Guerreiro, 2015 [62].	Portugal	To present and evaluate computer simulation as a learning tool	Evaluative survey (Likert scale and open-ended questions)	Seven qualified pharmacists	Virtual Human	Automated marking/grading<br><br>Immediate detailed feedback<br><br>Single user<br><br>In more than one pharmacy setting<br><br>Support sharing scenarios<br><br>Interactive animated avatar and preselected options.
Curtin, Finn, Czosnowski, Whitman, & Cawley, 2011 [46].	USA	To assess the impact of the computer simulation on student knowledge during another mannequin-based exercise	Post simulation survey with questions included demographic information, computer simulation scores, and satisfaction-related questions.	Pharmacy undergraduates at one university	Micro Sim	Automated marking/grading<br><br>Immediate detailed feedback<br><br>Single user<br><br>In more than one pharmacy setting<br><br>Sharing scenarios – not supported<br><br>No info

Douglass, Casale, Skirvin, & DiVall, 2013 [68].	USA	To implement and assess the impact of computer simulation on students' knowledge and skills	Pre and post-test for evaluating clinical competence<br><br>Post-course evaluative survey. (Likert scale)	Pharmacy undergraduates at one university.	TheraSim	Automated marking/grading<br><br>Immediate detailed feedback<br><br>Single user<br><br>In one pharmacy setting<br><br>Support sharing scenarios<br><br>Video, Audio, and Imagery and preselected options
Fuhrman, Buff, Eaddy, & Dollar, 2001 [63].	USA	To present and implement a virtual patient database in teaching continuity of care	Students graded on their responses Knowledge exam.<br><br>Post-course evaluative survey. (Likert scale)	Pharmacy undergraduates at one university	Virtual Family	Need faculty marking/grading<br><br>Immediate detailed feedback<br><br>Multiple users<br><br>In one pharmacy setting<br><br>Sharing scenarios – not supported<br><br>Text-based
Hussein & Kawahara, 2006 [47].	USA	To promote active learning and knowledge	Knowledge assessed based on 1) the care plan and messages left on the telephone 2) Final knowledge exam Also,<br><br>Educators' evaluative Survey. (Likert scale)<br><br>Students' evaluative Survey.	Pharmacy undergraduates and six faculty members at one university	Computer telephony interactive voice response system (IVR) and a text-to-speech (TTS) system.	Need faculty marking/grading<br><br>Delayed detailed feedback<br><br>Single user<br><br>In one pharmacy setting<br><br>Sharing scenarios – not supported<br><br>Voice- telephone-based and Text-based

			(Likert scale and open-ended questions)			
Fens, Dantuma-Wering, & Taxis, 2020 [64].	Netherlands	To present and evaluate a computer simulation and assess a wide range of competencies	Students are assessed individually and as a pharmacy team.<br><br>(The pharmacy team, with the largest number of patients, wins the game).	Postgraduate pharmacy students at three universities (Groningen, Utrecht, and Leiden)	Pharmacy Game Previously known as "GIMMICS/ PharmG"<br><br>https://pharmacygame.education/ (Active)	Need faculty marking/grading<br><br>Immediate detailed feedback<br><br>Single user<br><br>In one pharmacy setting<br><br>Support sharing scenarios<br><br>Utilising Microsoft applications that allow sharing documents, audio, and video calls (Human to human) interaction
Lambertsen et al., 2016 [34].	UK	Present a computer simulator and assess its effectiveness as a learning method	Observation of use of virtual patient<br><br>Focus group	Pharmacy undergraduates at one university	PharmaComm	Automated marking/grading<br><br>Immediate detailed feedback<br><br>Single user<br><br>In one pharmacy setting<br><br>Sharing scenarios – not supported<br><br>Preselected options
Lim et al., 2020 [33].	Australia & Malaysia	To assess knowledge and perceptions among students using a computer simulator	Self-administered questionnaire and focus group	Pharmacy undergraduates at two campuses.	The Monash OSCE Virtual Experience (MOVE)	Automated marking/grading<br><br>Immediate detailed feedback<br><br>Single user<br><br>In one pharmacy setting

						Support sharing scenarios<br><br>Interactive animated avatar and Preselected options
J. Marriott, Styles, & McDowell, 2012 [31].	Australia	To present a computer simulator and the integration experience within teaching the curriculum	Post-course students' feedback survey for two consecutive years.<br><br>A student focus group discussion was conducted.<br><br>A comprehensive evaluation was undertaken of staff and student use of Pharmville.	Pharmacy undergraduates	Pharmville	Needs faculty marking/grading<br><br>Delayed detailed feedback<br><br>Single user<br><br>In one pharmacy setting<br><br>Support sharing scenarios<br><br>Include: Video, Audio, and Imagery (Web-based) and/or free-text input
Menendez et al., 2015 [39].	Brazil	To present and evaluate computer simulation in teaching	Post-course surveys and on-site observations were performed when possible.	Pharmacy undergraduates at one university.	PharmaVP	Needs faculty for marking/grading<br><br>Delayed detailed feedback<br><br>Single user<br><br>In more than one pharmacy setting<br><br>Sharing scenarios – not supported<br><br>Include: Video, Audio, and Imagery (Web-based) and/or free-text input
Park & Summons, 2013 [65].	Australia	To present a computer simulator and students' perceptions	Evaluative survey – Likert scale	Pharmacy undergraduates at three universities	The Virtual Pharmacy Patient	Needs faculty for marking/grading<br><br>Delayed detailed feedback<br><br>Single user

						In more than one pharmacy setting<br><br>Sharing scenarios – not supported<br><br>Include: Video, Audio, and Imagery (Web-based)
Taglieri, Crosby, Zimmerman, Schneider, & Patel, 2017 [48].	USA	To assess the effect of incorporating Computer simulation on student competence and confidence	Pre-survey, interim survey, post-survey (Likert scale and open-ended questions)<br><br>Performance in mock clinic	Pharmacy undergraduates at one university	Shadow Health Digital Clinical Experience (DCE)	Automated marking/grading<br><br>Immediate detailed feedback<br><br>Single user<br><br>In diff pharmacy settings + other health education<br><br>Sharing scenarios – not supported<br><br>Video, Audio, and Imagery Text-based.
Tenório da Silva, Daniel Pereira, André Mascarenhas de Oliveira Santos Silva, Rafaella Menéndez, Andrés Silva Santos, Cleverton dos de Lima Florentino Júnior, Isaias Felizardo Neves, Sabrina Joany Dósea, Marcos Barbosa Lyra, Divaldo Pereira., 2020 [49].	Brazil	To evaluate the effect of using computer simulation to improve pharmacy students' knowledge	questionnaires before and after using the software and the Facts on Aging Quiz.	Pharmacy undergraduate at two universities	The Virtual Patient for Geriatric Education (VIPAGE)	Needs faculty marking/grading<br><br>Delayed detailed feedback<br><br>Single user<br><br>In more than one pharmacy setting (focus is on handling geriatric cases)<br><br>Shareable scenarios – No information<br><br>Text-based

Villaume, Berger, & Barker, 2006 [42].	USA	To assess students' knowledge using computer simulation	Post assignment survey was conducted to collect feedback regarding students' experiences with the tool.<br><br>knowledge test (by number of multiple choice questions (MCQs) as part of the final examination to examine students' knowledge retention and efficiency of incorporating the tool.<br><br>Students script for VP - graded Final exam – Knowledge retention	Pharmacy undergraduates	The Auburn University Virtual Patient (AUVP) simulation.	Needs faculty marking/grading<br><br>Delayed detailed feedback<br><br>Single user<br><br>In more than one pharmacy setting<br><br>Sharing scenarios – not supported<br><br>Multimedia and Speech recognition
Barnett, Gallimore, Pitterle, & Morrill, 2016 [37].	USA	To evaluate computer simulation vs a paper case on student confidence and engagement.	Pre- and post-evaluation survey (Likert scale)<br><br>Subjective-objective assessment plan (SOAP)	Pharmacy undergraduates at one university	Case Scenario/Critical Reader (CSCR) Builder authoring tool	Needs faculty marking/grading<br><br>Delayed detailed feedback<br><br>Single user<br><br>In more than one pharmacy setting<br><br>Sharing scenarios – No information<br><br>Video, Audio, and Imagery and Text-based
Battaglia, Kieser, Bruskiewitz, Pitterle, & Thorpe, 2012 [50].	USA	To present the development, implementation, and assess the effectiveness of	Pre and post assessment<br><br>(Likert scale, multiple choice questions (MCQs)	Pharmacists (42) and pharmacy students at one university	Virtual patient training via the online Moodle platform.	Needs faculty marking/grading<br><br>Delayed detailed feedback

		computer simulation	and short answer questions)			(Multiple users)<br><br>In diff pharmacy settings + other health education<br><br>Support sharing scenarios<br><br>Text-based
Coons, Kobulinsky, Farkas, Lutz, & Seybert, 2018 [69].	USA	to evaluate the impact of a virtual EHR on learning efficiency	Pre- and post-surveys<br><br>Well as a clinical pharmacy note which summarised a problem list and treatment recommendations.	Pharmacy undergraduates at one university	Virtual EHR/DocuCare	Needs faculty marking/grading<br><br>Delayed detailed feedback<br><br>Single user<br><br>In diff pharmacy settings + other health education<br><br>Support sharing scenarios<br><br>Preselected options and/or free-text input
Ives, Tucker, & Trovato, 2020 [70].	USA	To measure students' confidence and assess their performance and perception using EHR technology.	Pre- and post-activity surveys As well as performance scores were compared between students that used an EHR vs last year's students who used a paper-based medication form.	pharmacy undergraduates at one university	EHR GO!<br><br>https://ehrgo.com/<br><br>(Active)	Needs faculty marking/grading<br><br>Delayed detailed feedback<br><br>Single and multi-user<br><br>In diff pharmacy settings + other health education<br><br>Support sharing scenarios<br><br>Static images, Text, and Patient notes (Web-based)

Active: There is an available presence of the simulator's website, which offers new updates and shows specialised interest in Pharmacy education.

Inactive: There is (NO) available presence of the simulator's website or (NO) available updates or shows (NO) specialised interest and/or availability of scenarios focusing on Pharmacy education.
